# Impact of changes in controlled drugs legislation on benzodiazepine receptor agonist prescribing in Ireland: a repeated cross-sectional study

**DOI:** 10.1007/s00228-020-03063-z

**Published:** 2021-01-07

**Authors:** Cathal A. Cadogan, Colin P. Bradley, Kathleen Bennett

**Affiliations:** 1 School of Pharmacy and Biomolecular Sciences, Royal College of Surgeons in Ireland, Dublin, Ireland; 2 Department of General Practice, University College Cork, Cork, Ireland; 3 Division of Population Health Sciences, Royal College of Surgeons in Ireland, Dublin, Ireland

**Keywords:** Benzodiazepines, Z-drugs, Prescribing, Primary care, Legislation

## Abstract

**Purpose:**

To examine the impact of new controlled drugs legislation introduced in May 2017 on benzodiazepine receptor agonist (BZRA) prescribing in Ireland.

**Methods:**

A repeated cross-sectional analysis was conducted using publically available monthly pharmacy claims data from the General Medical Services (GMS) database. The study population comprised all GMS-eligible individuals aged ≥ 16 years from January 2016 to September 2019. Monthly prevalence rates of individuals receiving BZRA prescriptions per 10,000 eligible population were calculated and trends examined over time. Segmented linear regression of prevalence rates was used to examine changes before and after introduction of the legislation stratified by gender and age groups. Regression coefficients (β) and 95% confidence intervals (CIs) for monthly change were calculated.

**Results:**

Pre-legislation (January 2016 to April 2017), there was a significant monthly decline in benzodiazepine prevalence rate (β = − 1.18; 95% CI − 1.84, − 0.51; *p* < 0.001) but no significant change in Z-drug prescribing. Post-legislation (May 2017 to September 2019), increases in prevalence rates were observed for benzodiazepines (β = 1.04; 95% CI 0.17, 1.92; *p* = 0.021) and Z-drugs (β = 1.04; 95% CI 0.26, 1.83; *p* = 0.010). Post-legislation trends showed increases in BZRA prevalence rates among the youngest subgroup (16–44 years), with variable changes in the middle-aged subgroup (45–64 years) and no changes in the oldest subgroup (≥ 65 years).

**Conclusions:**

This study indicates that introduction of new legislation had limited impact on BZRA prescribing on the main public health scheme in Ireland. Interventions targeting specific population subgroups may be required to achieve sustained reductions in prescribing.

**Supplementary Information:**

The online version contains supplementary material available at 10.1007/s00228-020-03063-z.

## Introduction

Benzodiazepines have a range of clinical indications, including anxiety and insomnia. However, the associated risks of dependence and withdrawal symptoms and the potential for harm (e.g. falls, fractures, cognitive impairment) are well recognised [1]. Z-drugs are a comparatively newer group of non-benzodiazepine hypnotics with similar risk profiles to benzodiazepines [2, 3]. In order to minimise the risk of adverse outcomes (e.g. dependence, withdrawal symptoms), prescribing guidelines recommend that prescriptions for benzodiazepines and Z-drugs, which are collectively referred to as benzodiazepine receptor agonists (BZRAs), should be limited to short-term use (≤ 4 weeks) [4]. Despite the known risks associated with their use, BZRAs are still commonly prescribed in healthcare settings worldwide, with sizeable proportions of individuals receiving long-term prescriptions [5–7]. For example, a recent analysis of prescriptions issued on the main public health scheme in Ireland found that approximately one third of patients issued BZRA prescriptions were receiving them on a long-term basis (> 3 months) [7].

The prescribing and use of these medications in Ireland have been the focus of national attention at various time points over the last 20 years. The Benzodiazepine Committee was established in 2000 due to concerns over long-term prescribing and use of BZRAs and the associated risks of dependence and misuse [8]. This national, multidisciplinary committee was tasked with examining existing prescribing practices and making recommendations to promote rational prescribing in Ireland and reduce inappropriate use, thereby maintaining the clinical value of BZRAs for legitimate needs in practice. In 2002, the Benzodiazepine Committee published a detailed report with various recommendations relating to the prescribing and monitoring of BZRA use in Ireland [8], as well as prescribing guidelines [9]. The Benzodiazepine Committee recommended that all Z-drugs be included under the national Misuse of Drugs Regulations and that consideration be given to extending existing handwriting requirements, then applied only to temazepam and flunitrazepam, to all BZRA prescriptions (e.g. details of the prescribed medication to be written in the prescriber’s own handwriting). Prescription handwriting requirements are intended to enhance prescriber oversight of prescriptions involving controlled drugs, such as benzodiazepines, and reduce the potential for prescriptions to be altered or forged.

Due to the lack of complete national dispensing claims data at the time, the immediate impact of the Benzodiazepine Committee report and guidelines could not be comprehensively assessed. A number of small-scale, localised evaluations indicated that these publications had little, or no, immediate impact on BZRA prescribing practices [10, 11]. The most recent study in Ireland indicated that some progress has been made in reducing benzodiazepine prescribing nationally [7]. However, these reductions have been offset, to an extent, by increased Z-drug prescribing, despite evidence of potential harms associated with both drug classes including an increased risk of falls [12].

In May 2017, new controlled drugs legislation was implemented in Ireland [13]. This legislation included specific provisions with direct implications for the prescribing of all controlled drugs including BZRAs, opioids and stimulants (e.g. lisdexamfetamine), as well as other less commonly used drugs (e.g. phenobarbitone, selegiline). The legislation addressed one of the Benzodiazepine Committee’s previous recommendations by extending the scope of the Misuse of Drugs Regulations to include zopiclone and zolpidem. The 2017 legislation introduced additional requirements for most BZRAs which were also assigned a new controlled drug schedule. Prescriptions for these medications must now specify the total quantity prescribed in words and figures, although there are no handwriting requirements. The extent of prescription handwriting requirements was reduced for temazepam and flunitrazepam to now only require details of the medication and the total quantity of supply to be written in the prescriber’s own handwriting. A summary overview of the prescription requirements under this new legislation is provided in Supplemental Table 1. Three years following the implementation of these new regulations, their impact on BZRA prescribing has yet to be assessed.

The aim of this study was to ascertain the impact of the Misuse of Drugs Regulations 2017 [13] on BZRA prescribing in Ireland using national administrative pharmacy claims data. The objectives were to examine whether changes had occurred in each of the following post-introduction of the regulations in 2017:

I. Prevalence of individuals receiving BZRA prescriptions across all age/gender groups
II. Quantity of supply on dispensed BZRA prescriptions per 1000 eligible population per day.

## Methods

### Study design and study population

A repeated cross-sectional analysis was conducted using aggregated level dispensing data obtained directly from the Irish Health Service Executive (HSE)-Primary Care Reimbursement Services (PCRS), which is responsible for reimbursing pharmacies for eligible claims made. The analysis was restricted to those eligible for the General Medical Services (GMS) scheme, the largest community drug scheme administered by the HSE-PCRS. Data were included on all GMS-eligible individuals aged ≥ 16 years in Ireland over the study period 1st January 2016–30th September 2019.

The GMS scheme provides free health services based on means testing and age (those > 70 years have higher means thresholds) [14]. For prescription medications, a monthly co-payment (€0.50 per item) was introduced in October 2010. This co-payment increased over time to €2.50 per item (subject to a limit of €25 per family per month) as of December 2013. As of March 2017, the co-payment was reduced from €2.50 to €2.00 per item for persons aged ≥ 70 years. This change was applied to the entire GMS population from January 2018 and remained in effect for the remainder of the study period. As of the end of 2018, the scheme covered 32.9% of the general Irish population [15]. However, as the scheme is means-tested with higher eligibility thresholds for persons over 70, it over-represents socially deprived and older members of the population. The pharmacy claims database contains basic demographic information and details on monthly dispensed medications, coded using the World Health Organization Anatomical Therapeutic Chemical (ATC) classification system, for each individual within the scheme [16]. As all data were provided by the HSE-PCRS at an aggregated level, ethical approval was not required.

### Data analysis

For this analysis, all BZRA prescriptions were identified using relevant ATC codes (i.e. antiepileptics (N03AE), anxiolytics (N05BA), hypnotics/sedatives (N05CD, N05CF)) [16] and subsequently extracted from the database. All BZRAs licensed for use during the study period were included in the analysis (Supplemental Table 2). Benzodiazepines were further grouped according to plasma half-life (t_1/2_) as either short-acting (t_1/2_ ≤ 24 hours) or long-acting (t_1/2_ > 24 hours) [17, 18].

Monthly prevalence rate per 10,000 eligible population was calculated as the number of people in receipt of at least one BZRA prescription divided by the GMS-eligible population aged ≥ 16 years in the same month and this rate was multiplied by 10,000. The eligible population was identified from annual HSE-PCRS reports and the online HSE-PCRS GMS eligibility reporting tool across all study years (2016–2019) (available from: https://www.sspcrs.ie).

The total number of defined daily doses (DDDs) per prescription was calculated. DDDs were calculated for oral formulations of all BZRAs that were extracted from the database using standard reference values [16]. In addition to DDD, the diazepam milligram equivalent-DDD (DME-DDD) was also calculated [19]. This is a novel integrated unit of measurement that accounts for BZRA potency and enables population pharmacologic exposure to be estimated. In order to determine the DME-DDD for each drug, derived adjustment factors were used [19] which have been calculated by dividing the established DDD for individual drugs by the dose approximately equivalent to 10 mg of diazepam (Supplemental Table 2). Both DDDs and DME-DDDs per 1000 eligible population per day in each month were calculated by dividing the total number of DDDs or DME-DDDs by the number of days per month and eligible population (× 1000) for all included drugs. Monthly prevalence and DDDs/DME-DDDs per 1000 eligible population per day of supply were compared across gender and age groups (i.e. 16–44, 45–64, ≥ 65 years) to examine differences according to demographics.

### Statistical analysis

Segmented linear regression analyses [20] were used to examine changes in the trends over time (trends before and after introduction of legislation) for BZRAs following introduction of the Misuse of Drugs Regulations 2017 [13]. Level changes were not examined as prescriptions issued before the legislation came into effect in May 2017 remained valid, and therefore, an immediate, detectable impact on prescribing was not expected. The following intervals were used as the segments for the analyses: January 2016–April 2017 (pre-legislation) versus May 2017–September 2019 (post-legislation).

Segmented linear regression of prevalence rates was used to examine the monthly pre-slope and post- to pre-slope change, with regression coefficients (β) and 95% confidence intervals (CIs) presented. Segmented linear regression of DDDs/DME-DDDs per eligible population per day, and regression coefficients (95% CIs) for the underlying pre-slope and change post-slope are presented. Simple autocorrelation (lag-1) can be detected with the use of the Durbin Watson statistic. In the presence of significant autocorrelation, adjustment was made to the segmented regression analyses to account for this first-order serial autocorrelation (using PROC AUTOREG in SAS) which arises because observations taken over time are usually correlated.

Sensitivity analyses were performed to account for the potential lag time between introduction of the legislation and implementation in clinical practice. This involved assigning one month post the date of introduction to the pre-implementation period. As above, simple lag-1 autocorrelation was examined and adjusted for accordingly. *p* values < 0.05 were considered significant. Data analyses were performed using SAS statistical software v.9.4 (SAS Institute, Inc. Cary, NC, USA).

## Results

**Fig. 1:**
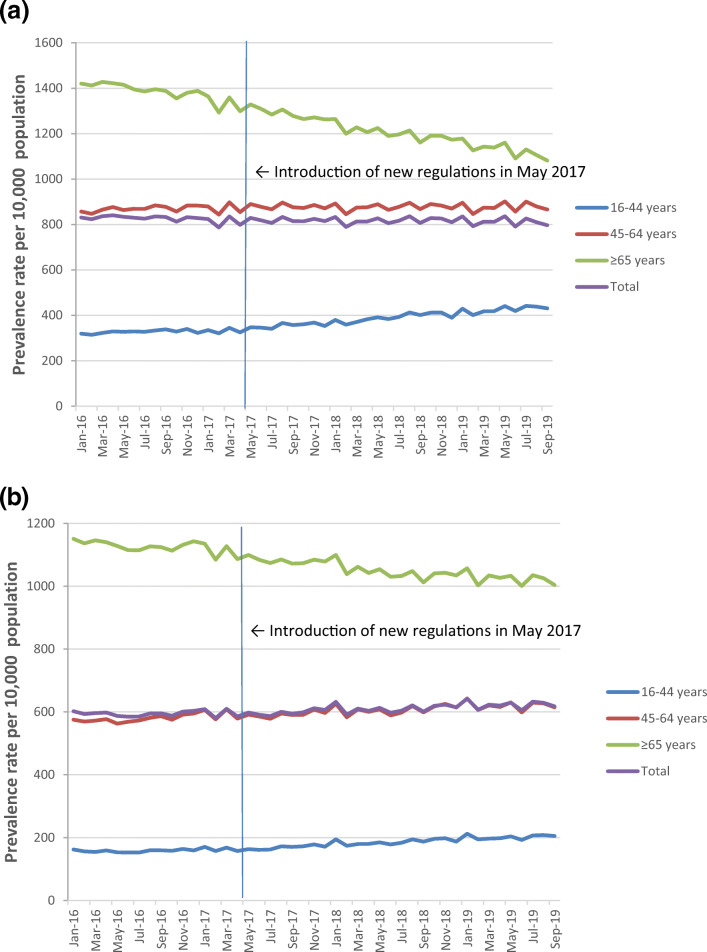
Prevalence of **a** benzodiazepine and **b** Z-drug prescriptions issued to GMS-eligible individuals aged ≥ 16 years before and after introduction of the new legislation

A total of 9,474,555 prescription claims for benzodiazepines (*n* = 5,518,691; 58.25%) and Z-drugs (*n* = 3,955,864; 41.75%) were processed for the patient cohort over the study period (January 2016 to September 2019). The monthly prevalence of benzodiazepine and Z-drug prescriptions dispensed on the GMS in January 2016 was 8.3% and 6.0%, respectively (Fig. 1).

### Trends in prevalence following Misuse of Drugs Regulations 2017

Prior to the legislation (January 2016 to April 2017), there was a significant monthly decline in the prevalence rate of benzodiazepine prescribing (β = -1.18; 95% CI -1.84, -0.51; *p* < 0.001) but no significant change in prevalence rate of Z-drug prescribing (β = 0.07; 95% CI − 0.53, 0.66; *p* = 0.82) (Fig. 1). Over the period following introduction of the legislation (May 2017 to September 2019), increases were observed in prevalence rates of benzodiazepine (β = 1.04; 95% CI 0.17, 1.92; *p* = 0.021) and Z-drug prescribing (β = 1.04; 95% CI 0.26, 1.83; *p* = 0.010) (Fig. 1). There were no differences in monthly prevalence trends when benzodiazepines were grouped as short-acting or long-acting (Supplemental Fig. 1).

#### Gender and age: benzodiazepines

The pre-intervention slope indicates that prior to introduction of the new legislation in May 2017, there was an increase in benzodiazepine prevalence rates from January 2016 to April 2017 in the younger (16–44 years, β = 1.01; 95% CI 0.61, 1.60; *p* < 0.001) and middle-aged groups (45–64 years, β = 1.00; 95% CI 0.26, 1.75; *p* = 0.01) and a decline in the older age group (≥ 65 years, β = − 7.78; 95% CI − 9.01, − 6.56; *p* < 0.001) (Fig. 1a). Similar trends were observed when the age groups were further stratified according to gender.

The change in slope following introduction of the legislation indicates that an increase in benzodiazepine prescribing was sustained in the younger age group (β = 2.46; 95% CI 1.81, 3.12; *p* < 0.001), whereas a decline in the pre-intervention slope was observed in the middle-aged group (β = -0.99; 95% CI -1.97, -0.009; p = 0.048) and no change in slope was observed in the older age-group (β = -0.04; 95% CI -1.66, 1.57; p = 0.96). Similar trends were observed when the age groups were further stratified according to gender.

#### Gender and age: Z-drugs

Prior to introduction of the new legislation in May 2017, there was an increase in Z-drug prevalence rates from January 2016 to April 2017 (pre-legislation) for the younger (16–44 years, β = 0.55; 95% CI 0.22, 0.88; *p* = 0.002) and middle-aged population age groups (45–64 years, β = 1.38; 95% CI 0.68, 2.08; *p* < 0.001) and a decline for the older age group (≥ 65 years, β = − 3.39; 95% CI − 4.71, − 2.07; *p* < 0.001) (Fig. 1b). Similar trends were observed when the age groups were further stratified according to gender.

The change in slope following introduction of the legislation indicates no consistent trends in Z-drug prescribing across the different age groups; an increase in slope post-legislation was observed in the younger age group (β = 1.01; 95% CI 0.57, 1.45; *p* < 0.001) and no change in slope was observed in the middle-aged (β = − 0.19; 95% CI − 1.11, 0.73; *p* = 0.68) and older age groups (β = 0.27; 95% CI − 1.48, 2.01; *p* = 0.76). Similar trends were observed when the age groups were further stratified according to gender.

### Quantity of supply per 1000 eligible population per day

**Fig. 2:**
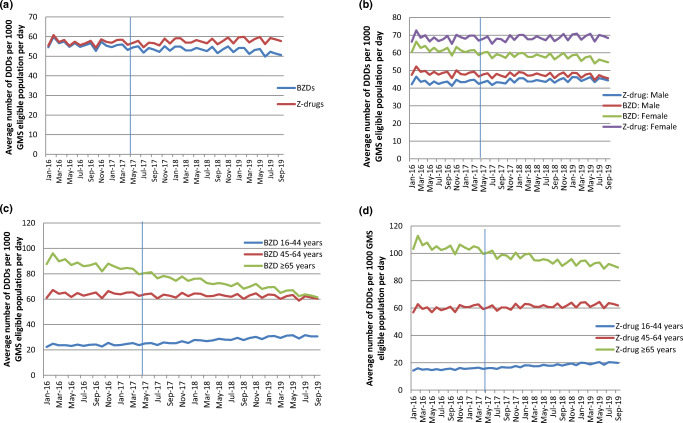
Average number of defined daily doses (DDDs) per 1000 GMS-eligible population per day before and after introduction of the new legislation (**a** = total population; **b** = population stratified according to gender; **c** = benzodiazepine (BZD) population stratified according to age; **d** = Z-drug population stratified according to age)

Table 1 provides an overview of observed trends in DDDs issued on BZRA prescriptions for GMS-eligible individuals aged ≥ 16 years before and after introduction of the new legislation. Prior to the legislation (January 2016 to April 2017), there was a significant decline in monthly DDDs per benzodiazepine prescription but no significant change in monthly DDDs per Z-drug prescription (Fig. 2). Over the period following introduction of the legislation (May 2017 to September 2019), no changes in the trends were observed in monthly DDDs per prescription for benzodiazepines or Z-drugs.

**Table 1:** Trends in defined daily doses per benzodiazepine receptor agonist prescription issued to GMS-eligible individuals aged ≥ 16 years before and after introduction of the new legislation (regression coefficients and 95% confidence intervals are presented for pre-intervention and change in post-intervention vs pre-intervention slopes; adjusted for first order autocorrelation)

Defined daily doses per benzodiazepine prescription						
	Pre-intervention			Change in post- compared to pre-intervention		
	Parameter estimate	95% CI		Parameter estimate	95% CI	
Overall trend	− 0.180**	− 0.257	− 0.103	0.099	− 0.003	0.201
By gender and age analysis						
Male	Parameter estimate	95% CI		Parameter estimate	95% CI	
16–44 years	0.109**	0.046	0.171	0.243***	0.160	0.325
45–64 years	− 0.008	− 0.100	0.083	− 0.063	− 0.184	0.058
≥ 65 years	− 0.561***	− 0.637	-0.484	0.069	-0.032	0.170
Female	Parameter estimate	95% CI		Parameter estimate	95% CI	
16–44 years	0.0528**	0.015	0.090	0.129***	0.080	0.178
45–64 years	− 0.039	− 0.122	0.045	− 0.088	− 0.171	0.049
≥ 65 years	− 0.883***	− 1.028	− 0.738	− 0.141	− 0.050	0.332
Defined daily doses per Z-drug prescription						
	Pre-intervention			Change in post- compared to pre-intervention		
	Parameter estimate	95% CI		Parameter estimate	95% CI	
Overall trend	− 0.043	− 0.124	0.038	0.107	0.000	0.214
By gender and age analysis						
Male	Parameter estimate	95% CI		Parameter estimate	95% CI	
16–44 years	0.067**	0.032	0.101	0.117***	0.072	0.163
45–64 years	0.071	− 0.003	0.145	0.003	− 0.094	0.100
≥ 65 years	− 0.352***	− 0.466	− 0.237	0.030	− 0.121	0.181
Female	Parameter estimate	95% CI		Parameter estimate	95% CI	
16–44 years	0.073***	0.043	0.103	0.058**	0.019	0.098
45–64 years	0.078	− 0.029	0.185	− 0.010	− 0.151	0.131
≥ 65 years	− 0.509***	− 0.677	− 0.340	0.086	− 0.136	0.307

*p < 0.05;**p < 0.01;***p < 0.001

Significant increases in monthly DDDs per benzodiazepine prescription were observed post-legislation for the younger age group across both genders, whereas no change in the trend was observed in the middle-aged and older age groups (Fig. 2). Similar trends in monthly DDDs per Z-drug prescription were observed post-legislation when stratified according to gender and age group (Fig. 2).

The analysis involving DME-DDD showed similar trends (Supplemental Table 3, Supplemental Fig. 2).

### Sensitivity analyses

Sensitivity analyses to account for the potential lag time between introduction of the legislation (January 2016–May 2017) and uptake into clinical practice (June 2017–September 2019) showed similar trends to those outlined above for both prevalence and quantity of supply when stratified according to gender and age groups (Supplemental Tables 4-5).

## Discussion

This study examined the impact of the Misuse of Drugs Regulations 2017 [13] on BZRA prescribing in Ireland using national pharmacy claims data for the GMS population aged ≥ 16 years. The findings indicate that the new legislation did not have the anticipated impact on the prescribing of these medications at a population level whereby overall prescribing of both benzodiazepines and Z-drugs increased marginally during the post-introduction period. Analysis of trends in prevalence rates over the period following introduction of the legislation showed increases in BZRA prescribing occurred in the youngest subgroup (16–44 years), with variable changes in the middle-aged subgroup (45–64 years; marginal decrease in benzodiazepine prescriptions; no change in Z-drug prescriptions) and no changes in trends for BZRA prescriptions issued to the oldest subgroup (≥ 65 years) from the pre-legislation decline. The lack of impact of the Irish legislation is in contrast to previous research. For example, an analysis of legislative changes in Ontario, Canada, that introduced additional measures targeting benzodiazepine prescriptions which included information recording and verification requirements by the relevant healthcare professionals, identified a small but statistically significant effect on prescribing rates [21]. However, as Z-drugs are not reimbursed on government-supported prescriptions in Ontario (with the exception of zopiclone in exceptional circumstances) and must, therefore, be paid for out-of-pocket, changes in Z-drug prescribing could not be assessed.

There are a number of plausible reasons for the regulations’ lack of observable impact on reducing the number of GMS-issued BZRA prescriptions in Ireland. Firstly, the stipulation for all BZRA prescriptions other than temazepam and flunitrazepam to specify the total quantity of supply in words and figures but without any handwriting requirements appears to have diminished any potential impact on the number of prescriptions issued. Secondly, of the two abovementioned benzodiazepines for which the most stringent prescription controls apply, only temazepam is currently licensed in Ireland. Our previous research has shown consistently larger declines in temazepam prescribing in Ireland over the years preceding the introduction of the regulations compared to other individual benzodiazepines for which less stringent prescription controls apply [7]. The rationale for maintaining tighter controls on temazepam prescriptions over other BZRAs is unclear as their introduction in the 1990s stemmed from concerns over the potential for misuse of temazepam and, in particular, the injection of the drug using liquid and gel-filled capsule formulations that were widely used at the time [22, 23]. However, these types of products are no longer available in Ireland. During the process of drafting the regulations, concerns were raised by professional representative bodies over the administrative burden of any extension of prescription handwriting requirements to all BZRA prescriptions. These were seen as outdated, unsafe and inefficient processes that were less amenable to detecting any unintended alterations to prescriptions compared to a printed version [24]. Legislation that focuses solely on the administrative aspect of how an individual prescription is written without adequate review of the underlying clinical need for the controlled drug is likely to be insufficient. This is supported by a recent study in the USA, whereby legislation that introduced mandatory reviews of patient records by prescribers in one state before issuing a controlled drug prescription led to a significant reduction in the average number of monthly benzodiazepine prescriptions [25].

Despite the legislation’s lack of observable impact at overall population level, stratification according to age-groups provided further insights into BZRA prescribing trends in Ireland before and after its introduction. Following introduction of the legislation, there was no change in the decline in BZRA prevalence rates among the older population observed pre-legislation. Although these findings could be viewed positively, it is important to note that a recent report highlighted that, compared to 15 other OECD countries, Ireland had the highest level of BZRA consumption among those aged ≥ 65 years [26]. Consequently, long-term use of BZRAs in the older population has been added to the 2019 list of national healthcare quality indicators [26]. Reducing long-term use of these medications in the older population, particularly benzodiazepines, has been promoted for many years through various prescribing criteria (e.g. Beers criteria [27, 28] and STOPP/START (Screening Tool of Older Person’s Prescriptions/Screening Tool to Alert doctors to Right Treatment) criteria [29, 30]) which may partly account for the observed trends. However, further targeted efforts are likely to be required to continue to reduce prescribing levels of the medications among the older population in Ireland.

Previous studies in a number of countries, including Ireland, have shown how reductions in benzodiazepine prescribing are offset by a shift towards increased Z-drug prescribing [7, 31, 32]. This was not observed in the current study. However, the study findings highlight an increasing prevalence of BZRA prescribing among the younger age groups, particularly those aged 16–44 years. Reported trends in BZRA prescribing among populations aged < 65 years are much less consistent, partly due to differences in definitions of age-groups across individual studies. However, analyses of BZRA prescribing in Canada [31] and several European countries [33, 34] have highlighted trends towards increasing levels of prescribing in younger age cohorts. This is of concern as, if prescribing among these age groups is not carefully monitored, it could ultimately perpetuate continued cycles of long-term BZRA use for future generations.

Previous research has highlighted a high prevalence of mental health disorders among young Irish adults, with anxiety disorders being one of the most common types [35]. An analysis of general practitioner (GP) records in Ireland identified that approximately 75% of mental health cases involved those aged < 65 years [36]. In most cases, patients were treated with medications (81%), including BZRAs, as opposed to receiving a psychological intervention (34%). It is beyond the scope of the current study to identify the exact reasons for the observed trends towards an increasing prevalence of BZRA prescriptions among the younger population groups. Adequate resourcing of mental health services has been a long-standing issue in Ireland which creates difficulties for those with mental health difficulties accessing relevant supports [37–39]. Some progress has been made with the introduction of the ‘Counselling in Primary Care’ service in 2013. This national service provides up to eight free counselling sessions within the primary care setting to GMS-eligible individuals aged ≥ 18 years with mild to moderate psychological difficulties [40]. GPs have reported numerous benefits to patients arising from this service (e.g. improved access to, and awareness, of talk therapy) [41]. However, an initial evaluation found that up to 24% of service users had to wait between 4 and 6 months to access counselling sessions [40]. The current study findings suggest a further consequence of these resourcing issues may involve an over-reliance on BZRA medications.

The higher level of BZRA consumption in the older aged population and women, as measured using DDDs, is consistent with previous research [42]. In addition to DDD, the DME-DDD metric was also used to assess population exposure. Despite having not been widely used in previous epidemiological studies to date, it has been proposed that it could make interpretation of population exposure estimates more meaningful by accounting for both consumption (DDD) and potency (DME) [19]. The DME-DDD findings showed higher levels of benzodiazepine exposure compared to DDD alone. The converse was true with Z-drugs on account of them having lower diazepam equivalence values compared to most benzodiazepines. A notable limitation of this approach is that the accuracy and precision of the diazepam equivalence values have not been fully established [19]. As the HSE-PCRS does not collect information on clinical outcomes, it was not possible to determine if differences in DME-DDD exposure resulted in differences in clinical outcomes or potential harms for GMS patients receiving BZRA prescriptions in Ireland.

The challenge in achieving sustained reductions in BZRA prescribing is further highlighted by the fact that, in addition to the legislative changes, guidelines on appropriate prescribing of BZRAs were also issued by the national HSE Medicines Management Programme in Ireland in February 2018 [43]. The analysis did not make specific attempts to examine the effects of these guidelines as they were non-statutory and, therefore, any impact on BZRA prescribing would have been more difficult to detect with the current study design. However, given the combination of the new legislation and prescribing guidelines, an overall reduction in BZRA prescribing would have been expected. Decisions regarding initiation, continuation and withdrawal of BZRA prescribing in primary care have been described as complex, demanding and uncomfortable [44]. The lack of impact of these recently introduced measures on BZRA prescribing in Ireland could indicate deficiencies with the legislative requirements in terms of prompting clinical reviews and relevant changes to BZRA prescriptions (as previously outlined above), as well a lack of effective dissemination and implementation and the underlying reasons for this need to be explored further.

Various policies and interventions targeting the prescribing and use of BZRAs have been examined to date with varying degrees of effect and often with a focus on the older population [45–48]. These include prescription monitoring programmes, reimbursement restrictions and brief interventions delivered in primary care. It remains to be seen how applicable and impactful some of these initiatives would be for the younger age groups in Ireland amongst whom BZRA consumption appears to be increasing. At present, the optimal approach to improving prescribing of these medications is unclear. It has been proposed that in order for sustainable changes in BZRA prescribing to be achieved, a combination of patient education and improved access to non-pharmacological therapies are also needed [45]. Irrespective of which approaches are used, it is imperative that a balance is struck between promoting deprescribing of inappropriate or unnecessary BZRA use and maintaining access to the medications for patients with legitimate needs. Future research should look to include perspectives of both patients and healthcare professionals in developing interventions and policies to improve BZRA prescribing and use in Ireland.

The study’s main strength is that it provides the first comprehensive assessment of any national measures introduced to target BZRA prescribing in Ireland. The analysis is based on the single largest and most comprehensive available prescription database in Ireland which provided a large population size and accurate information on dispensed medications over a wide time interval. The data also represent the majority of those aged over 70 years [14]. In terms of study limitations, it must be noted that the GMS pharmacy claims database is representative of approximately one-third of the Irish population and individuals with a lower socioeconomic status, women and older age are currently over-represented [14]. The data were provided by the HSE-PCRS at an aggregated level, not individual level; therefore, it was not possible to analyse changes in the incidence rates of new or long-term BZRA prescriptions. There was no electronic prescribing system in primary care in Ireland at the time of the study and, therefore, the pharmacy claims data do not contain information on clinical indications or patient outcomes. Finally, we did not examine whether changes in BZRA prescribing may have been offset by changes in other psychotropic medications. This is an area that should be explored in future research.

## Conclusions

This study indicates that the introduction of new legislation had limited impact on BZRA prescriptions issued on the main community drug scheme in Ireland. Policies and interventions targeting specific population subgroups may be required if sustained reductions in prescribing are to be achieved, including education around the long-term effects of BZRAs to reduce initiation and promote deprescribing. It remains to be seen to what extent, if any, observed changes are offset by changes in the prescribing of other psychotropic medication.

## Supplementary Information

ESM 1 (DOCX 96 kb)

## Data Availability

The data that support the findings of this study are available from the corresponding author upon reasonable request.
