# Supplementary material for: Impact of changes in controlled drugs legislation on benzodiazepine receptor agonist prescribing in Ireland: a repeated cross-sectional study
Source: Eur J Clin Pharmacol. 2021 Jan 7;77(6):903–12. doi: 10.1007/s00228-020-03063-z (PMC8128812; doi:10.1007/s00228-020-03063-z)
Supplement: Supplementary file 1 — (DOCX 96 kb) [file 228_2020_3063_MOESM1_ESM.docx]

## Supplementary material

**Supplemental Table 1** Overview of key requirements for prescriptions issued under the Misuse of Drugs Regulations 2017

| **Legal requirements*** | **Schedule 2 and 3**  **(applies to flunitrazepam and temazepam)** | **Schedule 4 Part 1**  **(applies to all other benzodiazepines and Z-drugs)** |
| --- | --- | --- |
| Be written in ink/indelible | Yes | Yes |
| Include prescriber’s full name (including first name**) | Yes | Yes |
| Include prescriber’s registration type | Yes | Yes |
| Include prescriber’s registration number** | Yes | Yes |
| Include date of issue and prescriber’s signature | Yes | Yes |
| Include prescriber’s address | Yes | Yes |
| Include prescriber’s telephone number | Yes | Yes |
| Include patient’s name (including first name**) and address | Yes [no longer needs to be handwritten**] | Yes |
| Include the name of the controlled drug | Yes [in prescriber’s own handwriting] | Yes [no handwriting requirements] |
| Include the dose, form and strength of the controlled drug | Yes [in prescriber’s own handwriting] | Yes [no handwriting requirements] |
| Specify the total quantity to be supplied (in both words and figures) | Yes [in prescriber’s own handwriting] | Yes [no handwriting requirements]** |
| Repeat prescribing is permitted | No | Yes |
| Prescription must be dispensed within 14 days of the date of issue | Yes | No |
| Prescription must not be dispensed before the date of issue on the prescription | Yes | Yes |
| Prescriber must have an address within the State | Yes | Yes |
| Emergency supply of the medication allowed | No | No |

Adapted from: <https://www.thepsi.ie/Libraries/Practice_Guidance/PSI_and_Medical_Council_Joint_guidance.sflb.ashx>

* Further details of specific exemptions to the regulations available from reference source

** New/amended requirement

**Supplemental Table 2** ATC codes, defined daily doses, diazepam milligram equivalence values and derived adjustment factors for prescribed benzodiazepines and Z-drugs

| **Benzodiazepine** | **ATC code** | **Duration of action**^**^ ([1](#_ENREF_1), [2](#_ENREF_2)) | **Defined daily dose (mg; oral route)** | **Equivalence to diazepam 10 mg (mg)** | **Adjustment factor (**[**3**](#_ENREF_3)**)** |
| --- | --- | --- | --- | --- | --- |
| [alprazolam](https://www.whocc.no/atc_ddd_index/?code=N05BA12&showdescription=yes) | N05BA12 | Short-acting | 1 | 0.5 | 2 |
| [bromazepam](https://www.whocc.no/atc_ddd_index/?code=N05BA08&showdescription=yes) | N05BA08 | Short-acting | 10 5–6 1.66–2 | 10 5–6 1.66–2 | 10 5–6 1.66–2^*^ |
| [chlordiazepoxide](https://www.whocc.no/atc_ddd_index/?code=N05BA02&showdescription=yes) | N05BA02 | Long-acting | 30 | 25 | 1.2 |
| [clobazam](https://www.whocc.no/atc_ddd_index/?code=N05BA09&showdescription=yes) | N05BA09 | Long-acting | 20 | 20 | 1 |
| [clonazepam](https://www.whocc.no/atc_ddd_index/?code=N03AE01&showdescription=yes) | N03AE01 | Long-acting | 8 | 0.5 | 16 |
| [diazepam](https://www.whocc.no/atc_ddd_index/?code=N05BA01&showdescription=yes) | N05BA01 | Long-acting | 10 | 10 | 1 |
| [flurazepam](https://www.whocc.no/atc_ddd_index/?code=N05CD01&showdescription=yes) | N05CD01 | Long-acting | 30 | 15–30 | 1-2^*^ |
| [lorazepam](https://www.whocc.no/atc_ddd_index/?code=N05BA06&showdescription=yes) | N05BA06 | Short-acting | 2.5 | 1 | 2.5 |
| [lormetazepam](https://www.whocc.no/atc_ddd_index/?code=N05CD06&showdescription=yes) | N05CD06 | Short-acting | 1 | 1–2 | 0.5–1^*^ |
| [midazolam](https://www.whocc.no/atc_ddd_index/?code=N05CD08&showdescription=yes) | N05CD08 | Short-acting | 15 | NA | NA |
| [nitrazepam](https://www.whocc.no/atc_ddd_index/?code=N05CD02&showdescription=yes) | N05CD02 | Long-acting | 5 | 10 | 0.5 |
| [prazepam](https://www.whocc.no/atc_ddd_index/?code=N05BA11&showdescription=yes) | N05BA11 | Long-acting | 30 | 10–20 | 1.5–3^*^ |
| [temazepam](https://www.whocc.no/atc_ddd_index/?code=N05CD07&showdescription=yes) | N05CD07 | Short-acting | 20 | 20 | 1 |
| [triazolam](https://www.whocc.no/atc_ddd_index/?code=N05CD05&showdescription=yes) | N05CD05 | Short-acting | 0.25 | 0.5 | 0.5 |
| **Z-drug** | **ATC code** | **Duration of action**^**^ ([1](#_ENREF_1), [2](#_ENREF_2)) | **Defined daily dose (mg; oral route)** | **Equivalence to diazepam 10 mg (mg)** | **Adjustment factor** |
| Zolpidem | N05CF02 | Short-acting | 10 | 20 | 0.5 |
| Zopiclone | N05CF01 | Short-acting | 7.5 | 15 | 0.5 |

^*^ In cases where a range was reported for the adjustment factor, the lowest value was used

^**^Short-acting (t_1/2_ ≤24 hours); Long-acting (t_1/2_ >24 hours)

NA = not available due to insufficient source data

**Supplemental Table 3** Trends in diazepam milligram equivalent-defined daily doses (DME-DDDs) per benzodiazepine and Z-drug prescriptions issued to GMS‐eligible individuals aged ≥16 years before and after introduction of the new legislation

| **DME-DDDs per benzodiazepine prescription** | | | | | | |
| --- | --- | --- | --- | --- | --- | --- |
|  | **Pre-intervention** | | | **Post-intervention** | | |
|  | Parameter estimate | 95% CI | | Parameter estimate | 95% CI | |
| Overall trend | -0.169** | -0.268 | -0.070 | 0.121 | -0.009 | 0.252 |
| By gender and age analysis | | | | | | |
| Male | Parameter estimate | 95% CI | | Parameter estimate | 95% CI | |
| 16-44 years | 0.238*** | 0.137 | 0.338 | 0.297*** | 0.164 | 0.430 |
| 45-64 years | 0.029 | -0.098 | 0.155 | -0.111 | -0.277 | 0.055 |
| ≥65 years | -0.693*** | -0.797 | -0.590 | 0.119 | -0.017 | 0.256 |
| Female | Parameter estimate | 95% CI | | Parameter estimate | 95% CI | |
| 16-44 years | 0.129** | 0.068 | 0.191 | 0.160** | 0.080 | 0.241 |
| 45-64 years | 0.038 | -0.094 | 0.169 | -0.164 | -0.337 | 0.009 |
| ≥65 years | -1.046*** | -1.221 | -0.870 | 0.219 | -0.012 | 0.451 |
| **DME-DDDs per Z-drug prescription** | | | | | | |
|  | **Pre-intervention** | | | **Post-intervention** | | |
|  | Parameter estimate | 95% CI | | Parameter estimate | 95% CI | |
| Overall trend | -0.021 | -0.062 | 0.019 | 0.0535 | 0.000 | 0.107 |
| By gender and age analysis | | | | | | |
| Male | Parameter estimate | 95% CI | | Parameter estimate | 95% CI | |
| 16-44 years | 0.033** | 0.016 | 0.051 | 0.059*** | 0.036 | 0.081 |
| 45-64 years | 0.036 | -0.001 | 0.073 | -0.001 | -0.047 | 0.050 |
| ≥65 years | -0.176*** | -0.233 | -0.118 | 0.015 | -0.060 | 0.091 |
| Female | Parameter estimate | 95% CI | | Parameter estimate | 95% CI | |
| 16-44 years | 0.037*** | 0.021 | 0.052 | 0.029** | 0.009 | 0.049 |
| 45-64 years | 0.039 | -0.015 | 0.092 | -0.005 | -0.075 | 0.066 |
| ≥65 years | -0.254*** | -0.339 | -0.170 | 0.043 | -0.068 | 0.154 |

*p<0.05;**p<0.01;***p<0.001

**Supplemental Table 4** Overview of results of primary analysis of trends in prevalence rates and sensitivity analysis

|  | **Primary analysis** | **Sensitivity analysis applying 1 month lag** |
| --- | --- | --- |
|  | Jan 2016-April 2017 compared to May 2017-Feb 2018 | Jan 2016-May 2017 compared to June 2017-Feb 2018 |
| **BZD female** |  |  |
| **16-44 years** | **Regression coefficient (95% CI)** | **Regression coefficient (95% CI)** |
| Change in slope | 2.31 (1.45, 3.18)*** | 2.19 (1.36, 3.03)*** |
| **45-64 years** |  |  |
| Change in slope | -0.95 (-2.35, 0.46) | -0.90 (-2.25, 0.45) |
| **65+ years** |  |  |
| Change in slope | 0.19 (-1.83, 2.21) | 0.26 (-1.67, 2.20) |

| **BZD male** |  |  |
| --- | --- | --- |
| **16-44 years** | **Regression coefficient (95% CI)** | **Regression coefficient (95% CI)** |
| Change in slope | 2.68 (2.08, 3.28)*** | 2.51 (1.90, 3.12)*** |
| **45-64 years** |  |  |
| Change in slope | -1.17 (-1.87, -0.47)** | -1.13 (-1.80, -0.45)** |
| **65+ years** |  |  |
| Change in slope | -0.30 (-1.48, 0.89) | -0.21 (-1.35, 0.93) |

| **Z-drug female** |  |  |
| --- | --- | --- |
| **16-44 years** | **Regression coefficient (95% CI)** | **Regression coefficient (95% CI)** |
| Change in slope | 0.93 (0.40, 1.46)** | 0.90 (0.40, 1.41)*** |
| **45-64 years** |  |  |
| Change in slope | -0.24 (-1.28, 0.80) | -0.16 (-1.16, 0.84) |
| **65+ years** |  |  |
| Change in slope | 0.28 (-1.72, 2.27) | 0.41 (-1.50, 2.32) |

| **Z-drug male** |  |  |
| --- | --- | --- |
| **16-44 years** | **Regression coefficient (95% CI)** | **Regression coefficient (95% CI)** |
| Change in slope | 1.11 (0.71, 1.51)*** | 1.04 (0.65, 1.43)*** |
| **45-64 years** |  |  |
| Change in slope | -0.24 (-1.10, 0.61) | -0.18 (-1.00, 0.65) |
| **65+ years** |  |  |
| Change in slope | 0.30 (-0.98, 1.58) | 0.38 (-0.84, 1.60) |

|  | **Primary analysis** | **Sensitivity analysis applying 1 month lag** |
| --- | --- | --- |
|  | Jan 2016-April 2017 compared to May 2017-Feb 2018 | Jan 2016-May 2017 compared to June 2017-Feb 2018 |
|  | **Regression coefficient (95% CI)** | **Regression coefficient (95% CI)** |
| **Overall BZD change in slope** | 1.04 (0.17, 1.92)* | 0.96 (0.11, 1.81)* |
| **Overall Z drug change in slope** | 1.04 (0.26, 1.83)* | 1.01 (0.27, 1.76)** |

*p<0.05;**p<0.01;***p<0.001

**Supplemental Table 5** Trends in defined daily doses per benzodiazepine and Z-drug prescriptions issued to GMS‐eligible individuals aged ≥16 years before and after introduction of the new legislation – sensitivity analysis of 1-month lag

| **Defined daily doses per benzodiazepine prescription** | | | | |
| --- | --- | --- | --- | --- |
| Gender and age group | **Pre-intervention** | | **Post-intervention** | |
| Male | Parameter estimate | Standard error | Parameter estimate | Standard error |
| 16-44 years | 0.126** | 0.029 | 0.228*** | 0.040 |
| 45-64 years | -0.008 | 0.045 | -0.063 | 0.060 |
| ≥65 years | -0.562*** | 0.035 | 0.074 | 0.047 |
| Female | 0.126** | 0.029 | 0.228*** | 0.040 |
| 16-44 years | 0.060** | 0.017 | 0.125*** | 0.023 |
| 45-64 years | -0.047 | 0.038 | -0.052 | 0.052 |
| ≥65 years | -0.884*** | 0.066 | 0.149 | 0.089 |
| **Defined daily doses per Z-drug prescription** | | | | |
| Gender and age group | **Pre-intervention** | | **Post-intervention** | |
| Male | Parameter estimate | Standard error | Parameter estimate | Standard error |
| 16-44 years | 0.075*** | 0.016 | 0.111*** | 0.022 |
| 45-64 years | 0.068 | 0.034 | 0.008 | 0.046 |
| ≥65 years | -0.358*** | 0.052 | 0.040 | 0.071 |
| Female | Parameter estimate | Standard error | Parameter estimate | Standard error |
| 16-44 years | 0.075*** | 0.0135 | 0.058** | 0.0185 |
| 45-64 years | 0.072 | 0.049 | -0.001 | 0.067 |
| ≥65 years | -0.515*** | 0.0765 | 0.098 | 0.104 |

*p<0.05;**p<0.01;***p<0.001

← Introduction of new regulations in May 2017

**Supplemental Fig. 1** Prevalence rate per 10,000 GMS-eligible population for short-acting (t_1/2_ ≤24 hours) and long-acting (t_1/2_ >24 hours) benzodiazepine (BZD) prescriptions issued to GMS‐eligible individuals aged ≥16 years before and after introduction of the new legislation

**(A)**

**(C)**

**Supplemental Fig. 2** Average number of diazepam milligram equivalent-defined daily doses (DME-DDDs) per 1000 GMS-eligible population per day before and after introduction of the new legislation

[A= total population; B = population stratified according to gender; C= benzodiazepine (BZD) population stratified according to age; D = Z-drug population stratified according to age]

**(B)**

**(D)**

**Reference**

1. Ashton H. Benzodiazepines: how they work and how to withdraw (also known as The Ashton Manual): University of Newcastle, England; 2002 [Available from: Available from: <http://www.benzo.org.uk/manual/index.htm>. Accessed: 08/08/17.

2. Passaro A, Volpato S, Romagnoni F, Manzoli N, Zuliani G, Fellin R. Benzodiazepines with different half-life and falling in a hospitalized population: The GIFA study. Gruppo Italiano di Farmacovigilanza nell'Anziano. Journal of clinical epidemiology. 2000;53(12):1222-9.

3. Brandt J, Alkabanni W, Alessi-Severini S, Leong C. Translating Benzodiazepine Utilization Data into Meaningful Population Exposure: Integration of Two Metrics for Improved Reporting. Clin Drug Investig. 2018;38(7):565-72.
